# Neural Mechanisms of Working Memory Accuracy Revealed by Recurrent Neural Networks

**DOI:** 10.3389/fnsys.2022.760864

**Published:** 2022-02-14

**Authors:** Yuanqi Xie, Yichen Henry Liu, Christos Constantinidis, Xin Zhou

**Affiliations:** ^1^Department of Computer Science, Vanderbilt University, Nashville, TN, United States; ^2^Department of Biomedical Engineering, Vanderbilt University, Nashville, TN, United States; ^3^Neuroscience Program, Vanderbilt University, Nashville, TN, United States; ^4^Department of Ophthalmology and Visual Sciences, Vanderbilt University Medical Center, Nashville, TN, United States; ^5^Data Science Institute, Vanderbilt University, Nashville, TN, United States

**Keywords:** neuron, prefrontal cortex, short term memory, neurophysiology, deep learning

## Abstract

Understanding the neural mechanisms of working memory has been a long-standing Neuroscience goal. Bump attractor models have been used to simulate persistent activity generated in the prefrontal cortex during working memory tasks and to study the relationship between activity and behavior. How realistic the assumptions of these models are has been a matter of debate. Here, we relied on an alternative strategy to gain insights into the computational principles behind the generation of persistent activity and on whether current models capture some universal computational principles. We trained Recurrent Neural Networks (RNNs) to perform spatial working memory tasks and examined what aspects of RNN activity accounted for working memory performance. Furthermore, we compared activity in fully trained networks and immature networks, achieving only imperfect performance. We thus examined the relationship between the trial-to-trial variability of responses simulated by the network and different aspects of unit activity as a way of identifying the critical parameters of memory maintenance. Properties that spontaneously emerged in the artificial network strongly resembled persistent activity of prefrontal neurons. Most importantly, these included drift of network activity during the course of a trial that was causal to the behavior of the network. As a consequence, delay period firing rate and behavior were positively correlated, in strong analogy to experimental results from the prefrontal cortex. These findings reveal that delay period activity is computationally efficient in maintaining working memory, as evidenced by unbiased optimization of parameters in artificial neural networks, oblivious to the properties of prefrontal neurons.

## Introduction

Working memory, the ability to maintain information in mind over a period of seconds is a core cognitive function, essential for higher human faculties (Baddeley, 2012). The neural basis of working memory has been a matter of debate (Constantinidis et al., 2018; Lundqvist et al., 2018). By some accounts, persistent activity generated in the prefrontal cortex and areas connected to it represents the information held in memory and determines what the subject recalls (Qi et al., 2015; Riley and Constantinidis, 2016). However, alternative models of working memory have also been proposed, identifying the rhythmicity of neuronal discharges as the critical neural variable of memory maintenance (Miller et al., 2018), suggesting that information may be maintained without an increase in firing rate during the delay period of working memory tasks (Stokes, 2015), or placing the site of working memory activity in sensory areas rather than the prefrontal cortex (Sreenivasan et al., 2014).

Generation of persistent activity has been modeled as a continuous attractor by biophysically inspired network models that generate a bump (peak) of activity representing the stimulus to be remembered (Compte et al., 2000). Predictions of these models about how neuronal activity, variability, and correlation and how these relate to performance of working memory tasks are borne by neurophysiological data (Wimmer et al., 2014; Barbosa et al., 2020). It is unclear, however, whether underlying assumptions of bump attractor models are realistic and whether their simplified structure is truly compatible with the diversity and variability of real neuronal responses. Criticism abounds, therefore, about whether they constitute a realistic model of working memory (Lundqvist et al., 2018; Miller et al., 2018). Many empirical results observed in neurophysiological recordings are also often difficult to interpret in the context of the bump attractor (Qi et al., 2021).

A potential way of understanding the nature of computations performed by neural circuits is to rely on Deep Learning methods (Cichy and Kaiser, 2019; Yang and Wang, 2020). Convolutional neural networks have had remarkable success in artificial vision and the properties of units in their hidden layers have been found to mimic the properties of real neurons in the primate ventral visual pathway (Yamins and DiCarlo, 2016; Khaligh-Razavi et al., 2017; Rajalingham et al., 2018; Bashivan et al., 2019; Cadena et al., 2019). It is possible to directly compare the activation profile of units in the hidden layers of artificial networks with neurons in cortical areas (Pospisil et al., 2018). Deep learning models are thus being used to understand the development, organization, and computations of the sensory cortex (Yamins and DiCarlo, 2016; Rajalingham et al., 2018; Bashivan et al., 2019). Another class of artificial networks models, Recurrent Neural Networks (RNNs) has been used recently to model performance of cognitive tasks and to study cortical areas involved in cognitive function (Mante et al., 2013; Song et al., 2017). RNN units exhibit temporal dynamics resembling the time course of neural activity and can be trained to simulate performance of working memory and other cognitive tasks (Masse et al., 2019; Yang et al., 2019; Cueva et al., 2020; Kim and Sejnowski, 2021).

Although RNNs and other Deep Learning methods have received wide recognition, their use for studying the brain has not been without criticism. The title of a recent review article is telling: “If deep learning is the answer, what is the question?” (Saxe et al., 2021). Neural network models are often under-constrained and it is almost always possible to produce a model that mimics the activity of the brain in some respects. The value of such a result is limited. Artificial Neural Networks do provide ways to understand how networks of units implement certain computations, however, and can further generate insights and hypotheses that can then be tested experimentally. It has been postulated that structured neural representations necessary for complex behaviors emerge from a limited set of computational principles (Saxe et al., 2021). Uncovering such principles through the use of artificial neural networks would be of value for the study of working memory.

We were motivated therefore to approach the mechanisms of working memory maintenance by simulating neural activity in RNNs trained to simulate working memory tasks and compare the mechanisms and computations that emerge in them with the behavior of real neurons and with biophysically inspired networks, which have been used to model the activity of the prefrontal cortex. We were thus able to determine how RNN networks maintained information in memory and to understand what aspects of their structure and activity could be used to draw further inference about the generation of cognitive functions.

## Materials and Methods

### Design of Recurrent Neural Networks

**FIGURE 1:**
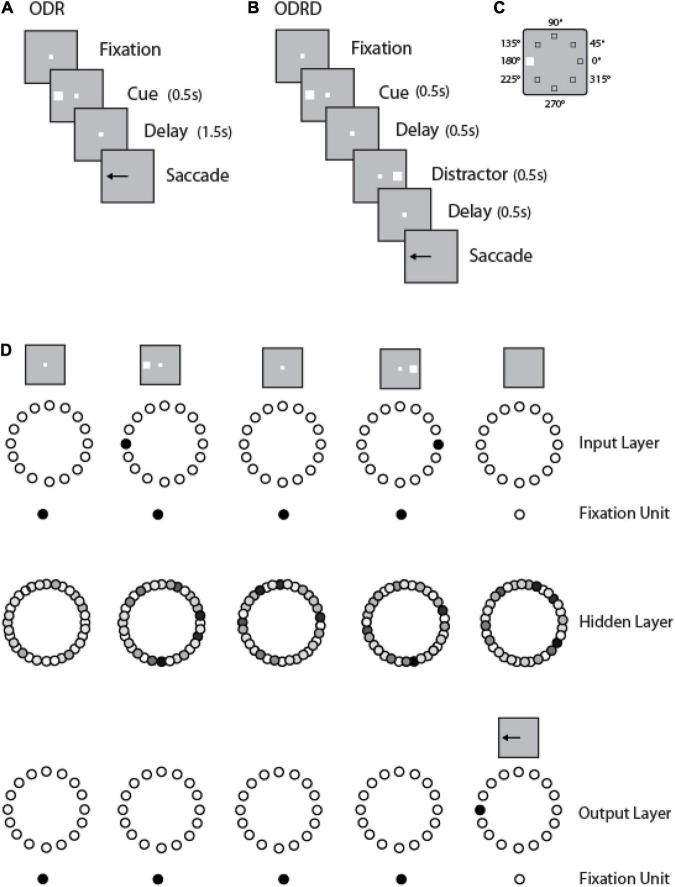
Working memory tasks and RNN approach. **(A)** Schematic Diagram represents the sequence of events in the ODR task. **(B)** Sequence of events in the ODRD task. **(C)** Possible locations of the cue (and distractor) presentation. **(D)** Schematic architecture of input, hidden, and output layers of the RNN network. Panels are arranged as to indicate successive events in time, in a single trial, across the horizontal axis. Appearance of the fixation point in the screen (top left panel) is simulated by virtue of activation of fixation units, a subset of the input units. Appearance of the visual stimulus to the left of the fixation activates input units in representing this location. Input units are connected to hidden layer units, and those to output layers units. In both tasks, the trained network generates a response to the remembered location of the cue by virtue of activation of the corresponding output unit.

We trained leaky RNNs to perform multiple working memory tasks: the Oculomotor Delayed Response or ODR task (Figure 1A); a variant of the ODR task requiring the subject to remember the location of a cue and ignore the presentation of a subsequent distractor (ODRD task in Figure 1B); and response inhibition tasks (variants of the antisaccade task), as we have described recently (Liu et al., 2021). We have found that the simultaneous training in multiple tasks facilitates acquisition of the working memory task and generalization across a number of task conditions. We then examined performance of the networks and activity their units generated in the working memory tasks.

Implementation was based in Python 3.8, using the TensorFlow package. The RNNs consisted typically of 256 recurrent units, with positive activity. The dynamics of the activity *r* of any unit were given by the following equation:


$$\tau \,\frac{d\,\text{r}}{d\,x}=-\text{r}+f\,\left(W^{r\,e\,c}\,\text{r}+W^{i\,n}\,\text{u}+\text{b}\,\sqrt{2\,\tau \,\sigma_{r\,e\,c}^{2}\,\xi }\right)$$

Here τ is the neuronal time constant (set to 100 ms in our simulations), **u** the input to the network, **b** the background input, *f* the neuronal non-linearity, ξ a vector of independent white noise process with zero mean and σ_*rec*_ the strength of noise (set to 0.05). This activity was discretized and each time step in our implementation represented 20 ms. That meant that a delay of 1.5 s was represented in 75 timesteps. We modeled the neuronal non-linearity based on the Softplus function


$$f\,\left(x\right)=\text{log}\,\left(1+e^{x}\right)$$

Output units, **z** read out the non-linearity from the network as:


$$z=g\,\left(W^{o\,u\,t}\,r\right),$$

where *g(x)* is the logistic function


$$g\,\left(x\right)=\frac{1}{1+e^{-x}}$$

and *W^out^* the weights of units connected to the output units.

Our networks received three types of noisy input: fixation, visual stimulus location, and task rule. The weights of the recurrent unit matrix (*W^rect^*) were initialized with random orthogonal initialization (Mezzadri, 2007), implemented with the scipy.stats.ortho_group function. Initial input weights (*W^in^*) were drawn from a standard normal distribution divided by the square root of the unit’s number. Initial output weights (*W*^*out*^) were initialized by the tf.get_variable function, using the default, Glorot uniform initializer, also known as the Xavier initializer (Glorot and Bengio, 2010). All weights could take either negative or positive values.

To train an RNN to perform the working memory tasks, we used a three-dimensional tensor as the input to the network that fully described the sequence of events. The first dimension of the tensor encodes the noisy inputs of three types: fixation, stimulus location, and task rule. Fixation input was modeled as a binary input of either 1 (meaning the subject needs to fixate) or 0, otherwise. The stimulus is considered to appear at a ring of fixed eccentricity, and its location is fully determined by the angular dimension. Stimulus inputs consisted of a ring of 8 units, with preferred directions uniformly spaced between 0 and 2π. For some simulations, a more fine-grained stimulus input was used; for those networks we increased the number of input units in a ring to 360 (while keeping fixed the number of 256 recurrent units in the network). The rule of the task was represented as a one-hot vector with a value of 1 representing the current task the subject is required to perform and 0 for all other possible tasks. The second dimension of the tensor encoded the batch size (number of trials). The third dimension encoded the time series for each trial.

A ring of 8 output units (plus one fixation output unit) similarly indicated the direction of gaze at each time point in the trial. Networks with 360 output units were also used, whenever the input unit number was increased. While the fixation point was on, the fixation output unit should produce high activity. Once the fixation input was off, the subject had to make an eye movement in the direction of the stimulus in the ODR task (and the direction of the first stimulus in the ODRD task), which was represented by activity in the network of tuned output units. The response direction of the network was read out using a population vector method. A trial is considered correct only if the network correctly maintained fixation (fixation output unit remained at a value > 0.5) and the network responded within 36° of the target direction.

An important consideration in the activity generated by RNN networks is the duration of the task epochs, and whether this is fixed or varies during training. Networks trained with fixed delay intervals tend to generate activity that rises and peaks at a certain time point in the trial; in contrast, networks trained with variable delays generate more stable persistent activity (Liu et al., 2021). We therefore trained networks with both fixed and variable delay period, the latter using training trials where the delay period could take a value between 0 and 3 s in 0.1 s increments. Once fully trained, these networks could still be tested with task epoch durations equal to those used in the experimental studies: the fixation epoch is the period before any stimulus is shown, and lasted for 1 s. The cue presentation epoch lasted for 0.5 s and was followed by the delay period (1.5 or 3.0 s).

The RNNs are trained with supervised learning, based on variants of stochastic gradient descent, which modifies all connection weights (input, recurrent and output) to minimize a cost function *L* representing the difference between the network output and a desired (target) output (Yang and Wang, 2020). We relied on the Adam optimization algorithm (Kingma and Ba, 2015) to update network weights iteratively based on training data. For each step of training, the loss is computed using a small number *M* of randomly selected training examples, or minibatch. Trials representing all six tasks were included in a single minibatch during training of our networks. Trainable parameters, collectively denoted as θ are updated in the opposite direction of the gradient of the loss, with a magnitude proportional to the learning rate η:


$$\Delta \,\theta =-\eta \,\frac{\partial L}{\partial \Theta }$$

We found that the ability of the networks to master the task was quite sensitive to the value of η. This was set to 0.001 for the simulations included in the paper.

The activity of recurrent units was read out at discrete time points representing 20 ms bins. These can be compared with “Peri-stimulus Time histograms” of real neurons. Firing rates of individual units and population averages are typically presented as normalized rates, obtained by subtracting the unit’s baseline firing rate (obtained in the 1 s period prior to the appearance of the cue) from the unit’s raw firing rate during the course of the trial, and dividing by the same baseline firing rate. Furthermore, we identified three stages of training, mid-trained (defined by a performance level of 35–65%), mature (achieving a performance level of 65–95%) and fully trained (achieving performance ≥ 95%).

### Quantification and Statistical Analysis

#### Saccade Endpoint Analysis

**FIGURE 2:**
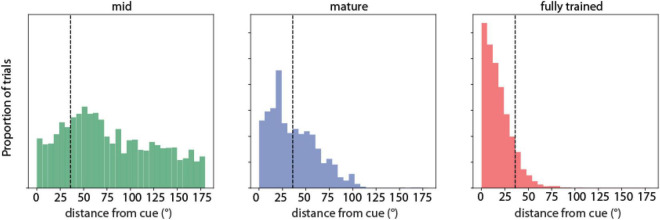
Saccadic endpoint analysis. Saccadic endpoint distributions to target stimulus locations (°) of RNN units responsive to the ODR task during three training stages (indicated as mid-trained, mature, and fully trained networks). *N* = 711 correct, 2,617 error trials for the mid-trained stage; *N* = 983 correct, 809 error trials in the mature stage; *N* = 5,101 correct, 659 error trials for the fully trained stage, respectively.

To visualize the difference in distance between the predicted saccade location from the RNN network and target stimulus position, the distribution of saccade location relative to the stimulus location was plotted during the three training stages for both correct and error trials. We refer to this as “saccadic endpoint” analysis in Figure 2.

#### Activity of Recurrent Neural Network Units

We trained RNN networks with the ODR (Figure 1A) task, which requires subjects to remember the spatial location of a stimulus and indicate that by shifting their eye position, and the ODRD task (Figure 1B), which adds a distractor stimulus that needs to be ignored between the presentation of the cue and response. We then determined the activity of RNN units during the delay period in the three training stages and in correct and error trials. We identified neurons that generated persistent activity as those whose activity during the delay period was significantly elevated over the fixation period (based on a *t*-test, evaluated at the *p* < 0.05 significance level). We constructed PeriStimulus-Time Histograms (PSTH) from different training stages, using both correct and error trials. Typically the best location of each neuron was identified, and then responses of multiple neurons were averaged together. We performed comparisons between conditions (e.g., correct vs. error trials) by averaging activity from all units that generated persistent activity during the task (typically in the order of 150–200 units per network instantiation), across multiple networks (typically 30 networks).

#### Network Peak of Activity

In order to visualize the spatial extend of RNN unit activation across the network, we created a two-dimensional heatmap of activity. For this analysis, simulations with 360 input and output units were used. During this ODR task, which has a 1.5 or 3.0 s delay time, we first sorted the units based on the location in which each unit achieved its peak firing rate during the delay period. We then generated a heatmap in ascending order where each row represents the firing rate of a single unit normalized by its preferred delay firing rate.

#### Behavior—Rate Correlation Analysis

In order to explore the correlation between mean firing rate and saccade deviation in network hidden units, the Pearson correlation coefficient was computed. We only used the last 1 s of the delay period in the ODR task as the neural activity which was most representative and sensitive right before the saccade. The firing rate deviation of each unit was computed as the difference between the actual firing rate in a trial and the median firing rate across all trials for the same cue location. The value of saccade deviation of each unit was the absolute difference between actual location and median of locations. The sign of saccade deviation was determined according to the tuning function of each unit. Saccade deviation toward the preferred stimulus had positive sign, while saccade deviation away from the preferred stimulus had negative sign. The firing rate and saccade deviations were then reordered based on the tuning locations and the correlation coefficients were computed. We observed the averaged correlation coefficients of spatial tuning locations and its distribution of delay neurons in the fully trained stage.

#### Fano Factor Analysis

Fano factor was computed as a measure of variability of RNN units in these simulations. For each unit that exhibited significantly elevated delay-period activity (as defined in the previous section) we computed the delay-period firing rate in the entire delay period, separately for each of the 8 ODR stimulus locations. We then repeated this calculation across 16 correct trials. The variance of this estimate, divided by the mean defines the Fano factor for each unit at each location. We then rotated the neural response to the stimulus at different spatial locations so that the best location of each unit was represented in the graph’s center location. The firing rate and Fano factor at each location were plotted as a function of distance from the unit’s preferred location to generate the average tuning curves and Fano factor plots. Here, we also used the last 1 s of delay period in the ODR task.

## Results

We trained RNN networks to perform variants of the Oculomotor Delayed Response task (ODR—Figure 1A), including a version of the task with a distractor (ODRD—Figure 1B). A cue stimulus could appear at one of eight locations arranged on a ring, thus deviating by 45° of angular distance relative to each other (Figure 1C). Subjects performing this task are required to maintain the stimulus in memory, and after a delay period, to perform an eye movement to its remembered location, ignoring any distractor stimulus, if one is present (Zhou et al., 2016). The RNNs simulated the task by receiving an input representing the cue location, representing its location in activity over a delay period of 1.5–3 s and generating a response corresponding to an output location on the ring (Figure 1D). This was computed by combining the activity of output units and could therefore vary continuously in the range of 0–360°. We analyzed the performance of the network in the task, the activity of units, and the relationship between the two.

### Task Performance

We first determined how the network performed the task and how performance changed as a result of training. We used the calculated position of the output units of the network, which corresponds to the endpoint of the saccade of subjects performing the ODR task, as the main metric of performance. Early in training, the RNN networks exhibited a near uniform distribution of responses, with saccadic endpoints covering all possible output positions. Only a small fraction of these trials was considered correct by our definition of falling within 36° of the cue position (Figure 2, left). As training progressed, the percentage of correct trials increased (Figure 2, middle). The distribution of error trials also markedly shifted, so that in the fully trained network errors deviated only slightly beyond the 36° criterion deviation value. A smaller peak in the error distribution of the mid-stage network corresponded to the location adjacent to the actual stimulus position (at 45° degrees relative to the cue). The skewness in the distribution was further exaggerated in the fully trained networks (Figure 2, right), however, even in this phase, RNNs generated a distribution of trials with variable accuracy, including error trials. That allowed us to determine how activity of neurons in the network related to behavioral performance.

### Recurrent Neural Networks Activation Compared to Attractor Models

**FIGURE 3:**
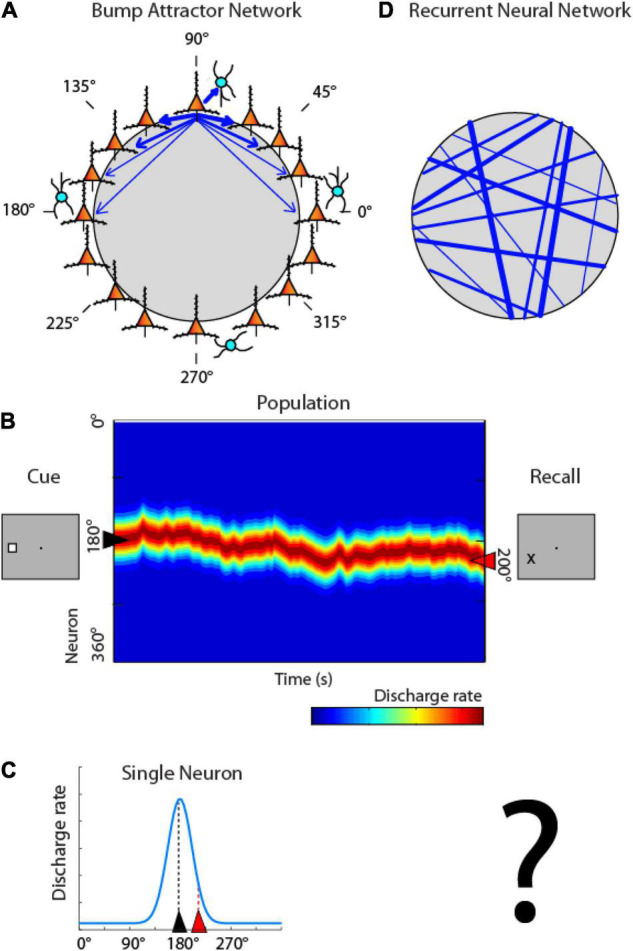
Bump attractor vs. RNN models. **(A)** Schematic diagram of the bump attractor model. Neurons representing different stimulus location are arranged in a ring so as to indicate each neuron’s preferred location. Synaptic connections between neurons with similar preference are stronger. **(B)** Schematic of the pattern of activity in the network. *Y* axis represents neurons in the ring with different spatial preference. A stimulus appearing at the left creates a peak of activity in the network centered on the 180° location. This peak may drift in time during the delay period of the task. The location of the peak at the end of the delay period determines the location that the subject recalls. **(C)** Activity of individual neurons is modulated based on the drift of activity in the network; a neuron with a peak at 180° may be maximally activated at the beginning of the delay period, but its activity is expected to be much lower at the end, if the peak has drifted. **(D)** Schematic diagram of the RNN networks. Units with different preference are arranged randomly in the network, and strengths of connections develop after training, with no geometric arrangement.

One class of models posits that working memory is mediated by the persistent activity of neurons in the prefrontal cortex (Riley and Constantinidis, 2016; Constantinidis et al., 2018), which behave as a continuous attractor. These are referred to as “bump attractor” models because the bump (peak) of activity across the network of neurons with different stimulus preferences ends up determining the location that the subject remembers (Compte et al., 2000; Wimmer et al., 2014). In this scheme, activity during the working memory interval of the task is maintained in the network by virtue of connections between neurons (Figure 3A). Neurons with different spatial tuning, i.e., maximally activated by stimuli appearing at different spatial locations on the screen can be thought of as forming a ring (hence “ring attractor” is another term for this type of network). Neurons with similar spatial tuning are strongly connected with each other (indicated by the width of arrows in Figure 3A). Appearance of a stimulus at one location, e.g., at the 180° location, to the left of the fixation point, maximally excites neurons on the ring representing that location. Activation then persists even after the cue is no longer present, by virtue of their pattern of connections between neurons that allow activity to reverberate in the network (Constantinidis and Wang, 2004). Maintenance of activity is not perfect in the network; the peak of activity may drift in time, resulting in behavioral inaccuracies. This is illustrated in Figures 3B, now representing the ring of neurons of Figure 3A in linear fashion, across the *y*-axis. The critical element of the model is that activity at the end of the delay period determines the location that the subject recalls (Figure 3B right). Activity of individual neurons can then be expected to be higher or lower depending on whether the bump of activity drifts toward the neuron’s preferred location or away from it (Figure 3C).

Artificial networks that simulate the bump attractor have been shown to accurately capture properties of prefrontal neurons (Compte et al., 2000; Wimmer et al., 2014; Barbosa et al., 2020). However, the pattern of connectivity between neurons active during working memory is generally unobservable and it is unknown if they represent a realistic depiction of neural circuits, shaped by activity. The pattern of connections between RNN units is unstructured initially and emerges during training (Figure 3D). Such simulations can allow us therefore to test the pattern of connectivity that emerges in networks after optimization and the relationship between delay period and behavior that develops.

**FIGURE 4:**
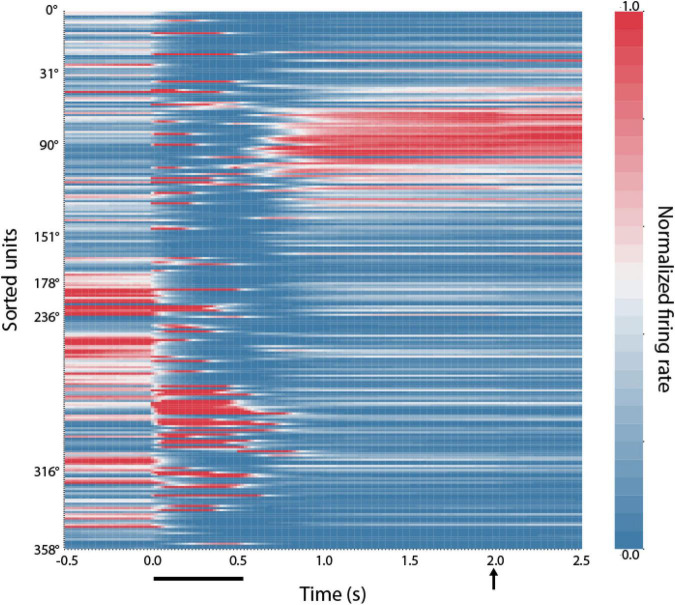
Location of RNN unit activation during working memory tasks. Firing rates of RNN units responsive to the ODR task during the mature-training stage. Each row represents activity of a single unit during presentation of the unit’s preferred stimulus in the ODR task. Units have been sorted based on the location where each unit achieves the peak firing rate during the delay period (indicated at the *y*-axis label). Color scale represents activity normalized by the baseline firing rate, separately for each neuron. Horizontal line at the bottom of the plot indicates time of cue appearance at time 0; vertical arrow, time of response.

We thus sought to examine how RNNs implement this task. Results of a typical network are shown in Figure 4. After training in the ODR task, individual RNN units exhibited a preferred stimulus location (they are shown arranged in ascending order across the ordinate of the plot, in a similar fashion as the model of Figure 3B). During trials involving presentation of the stimulus at the 90° location, units whose preferred delay period activity were near this location continued to be active through the delay period when no stimulus was present. This pattern of activity was reminiscent of bump attractor networks (Figure 3B), and experimental results (Funahashi et al., 1989).

**FIGURE 5:**
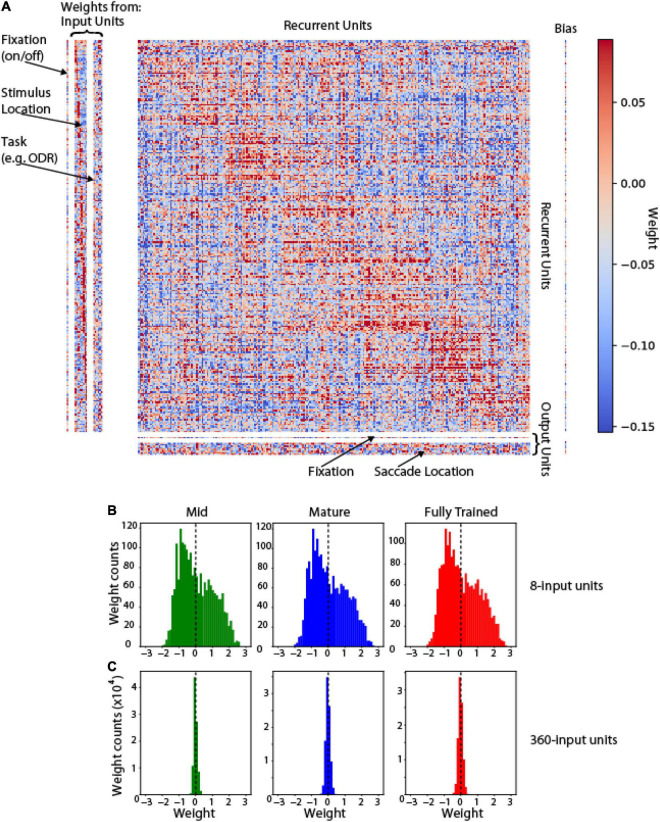
Weight Matrix. **(A)** Full connectivity matrix for a trained network in the ODR task. Left: weights between input and hidden (recurrent) units. Center: weights between recurrent units. Bottom: weights between recurrent and output units. Arrangement of recurrent units in the corresponding dimension of each matrix was obtained, by sorting them based on their preferred stimulus location in the ODR task. Red points indicate excitatory weights between corresponding units; blue points indicate inhibitory weights. **(B)** Weight distribution if input-to-recurrent unit weights in mid-, mature- and fully trained networks incorporating 8 input units (as in **A**). **(C)** Weight distribution in networks incorporating 360 input units.

It was also informative to understand how the pattern of connections between these units was shaped after training. Analysis of all unit weights in shown in Figure 5A. Units in the hidden (recurrent) layer with similar tuning were more likely to be connected to each other (positive weights cluster around the diagonal). This weight matrix that emerges after training recapitulates the weight structure of bump attractor models, in which the footprint of synaptic connections is directly dependent on tuning similarity (Compte et al., 2000; Wimmer et al., 2014). Changes in network unit number (e.g., increase of input units from 8 to 360) produce rescaling of weights in the course of training (Figures 5B,C).

**FIGURE 6:**
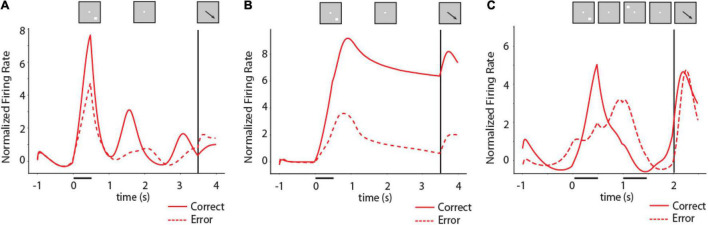
Activity of RNNs during working memory tasks. **(A)** Mean firing rate of RNN units responsive to the ODR task for the location corresponding to the best delay period activity of each unit in fully trained networks. Solid line indicates PSTH of correct trials, dashed line indicates PSTH of error trials. *N* = 2,704 units for correct trials, 244 units for error trials. Inset on top of figure indicates schematically the sequence of events in the task; location of stimulus used to construct the mean firing rate plot differed for each unit. **(B)** As in **(A)** for an RNN network trained with a variable delay period. *N* = 5,039 units for correct trials, 144 units for error trials. **(C)** Mean firing rate of RNN units responsive to the ODRD task. Solid line indicates PSTH of correct trials when cue is in the best location of each unit; dashed line indicates PSTH of error trials. *N* = 2,951 units for correct trials, 208 units for error trials.

The similarity of the RNN networks with the bump attractor model was not absolute. RNN units displayed considerable dynamics, with activity after the cue appearance that quickly decayed and reemerged later in the delay period (as in Figure 5A), or activity that ramped up slowly after the appearance of the stimulus and peaked in the delay period (as in Figure 5B—further discussed below). Furthermore, RNN units often exhibited different preferred location for the cue and delay periods. In the network instantiation shown in Figure 3 the stimulus appeared at the 90° location and units have been sorted based on their maximum delay-period activity. Only a few of the units active during the delay also show peaks of activation during the cue presentation, which typically subside and reemerge (as in Figure 6A). A second cluster of activation during the cue appearance at 90° was observed among units whose preferred delay period activity was near 270°. This finding clearly deviates from the behavior of bump attractor networks whose peak of cue activation persists stably into the delay period but is in fact more similar to experimental data from prefrontal neurons that often exhibit different preferences at different task epochs (Rao et al., 1999; Spaak et al., 2017).

### Unit Responses and Relation With Behavior

To appreciate better the full time-course of activation of RNN units in the task, we plotted PSTHs of individual units (Figure 6). Different RNN units generated activity at various times during the task, including in the delay period. As we have shown recently (Liu et al., 2021), the time course of activation in RNN units varies considerably depending on the timing of task events. Networks trained with fixed delay intervals are much more likely to produce activity that peaks at specific times during the task (Figure 6A). In contrast, networks trained with a variable delay period can generate stable delay period activity that remained at a high level until the response period (Figure 6B).

Our first objective regarding the relationship of RNN activity and behavior was to determine whether the delay period activity generated by RNN units during working memory tasks determined recall, in analogy with the bump attractor models and experimental results from the prefrontal cortex. This was indeed the case. The activity of RNN units in the delay period of the task was predictive of the behavior of the network, in multiple ways. Firstly, error trials were characterized by lower levels of activity following a unit’s preferred delay-period location in the ODR and ODRD tasks. Mean activity in the last 1 s of the delay period following the preferred location of each RNN unit was significantly higher in correct than error trials for networks trained with a fixed 3 s delay, as in Figure 5A (two-tailed *t*-test, *t*_2_,_946_ = 2.48, *p* = 0.013). The difference was much more pronounced for networks trained with a variable delay period as in Figure 5B (two-tailed *t*-test, *t*_5_,_181_ = 18.9, *p* = 2.1 × 10^–58^). We based this analysis exclusively on activity of units that generated elevated delay period activity, pooled across multiple network instances. The variability of individual units was very subtle from trial-to-trial (discussed in more detail in section “Unit Variability,” below). The difference between error and correct trials was mostly the result of differences between network instances. In other words, networks in which units did not generate high levels of delay period activity were more likely to generate errors.

In the ODRD task (Figure 6C), the critical comparison was that of activity in the second delay interval of the task, following a cue in the unit’s preferred location. Mean RNN unit activity in this interval was higher in correct rather than error trials (two-tailed *t*-test, *t*_3_,_157_ = 7.3, *p* = 6.7 × 10^–13^). These results mirror findings in neurophysiological experiments: prefrontal neurons exhibit reduced firing rate for their preferred stimulus during the delay period of error trials in the ODR (Funahashi et al., 1989; Zhou et al., 2013) and ODRD tasks (Zhou et al., 2016).

**FIGURE 7:**
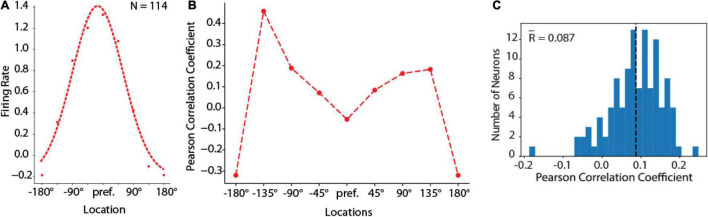
Behavior-rate correlation analysis. **(A)** Population tuning curve from units with delay period activity in the fully trained stage of the ODR task constructed by rotating neuron responses to the stimulus presentation at different spatial locations so that the best location of each neuron is represented in the graph’s center location. Left curve represents the best Gaussian fit. Curve represents average of *N* = 114 neurons, averaged over 16 correct trials for each location. **(B)** Averaged Pearson Correlation coefficients between delay firing rate deviation and saccade deviation at spatial tuning locations of RNN delay units during the fully trained stage, for the same population of neurons shown in **(A)**. **(C)** Distribution of Pearson correlation coefficients between delay firing rate deviation and saccade deviation of RNN units that exhibited delay period activity (*N* = 114 neurons).

Secondly, the behavioral outputs of the RNN networks (which we refer to as “saccadic endpoints” in analogy to the eye movements generated by subjects in the ODR task) were related to the mean activity of individual units. For different deviations of saccadic endpoints from their mean position, activity in individual units showed a deviation in the direction predicted by the unit’s tuning function, not unlike what has been reported for prefrontal neurons in the context of the bump attractor model (Figure 3C). For this analysis we rotated the tuning of each neuron so that the best location is represented at the center of the tuning curve. Let’s consider a trial involving stimulus appearance at the flank of the neuron’s tuning curve, at +45° degrees from the peak. If the delay period activity of this unit contributes causally to the recall of the stimulus, then on trials when the activity of this unit was higher than average we would expect the saccadic endpoint to deviate in the direction of the unit’s preferred location; on trials when its activity is lower than average, we would expect the saccadic endpoint to deviate in the opposite direction. The correlation of any single unit with behavior would be expected to be small (since behavior is determined by the simultaneous activation of hundreds of units) but positive. This is the result predicted by the bump attractor and validated in prefrontal recordings (Wimmer et al., 2014). This was precisely the case in our RNN simulations, as well (Figure 7). A small but significant positive correlation was observed between the unit’s delay period activity and the endpoint of the saccade. Across the population of units, firing rate deviations from tuning curve correlated positively with saccade deviations from median saccade position (Figure 7C). The mean correlation value (*r* = 0.087) was significantly higher than 0 (one-sample *t*-test, *p* = 1.07 × 10^–25^). Importantly, this positive relationship held for locations in the flanks of the unit’s receptive field (locations ± 45–135 in Figure 7B). For the peak and tail of the unit’s tuning curve no such relationship would be expected (as deviation of saccadic endpoint from either direction of the peak would be expected in lower firing rate, and no net positive correlation), resulting in an “M”-shaped averaged correlation at tuning locations.

### Unit Variability

**FIGURE 8:**
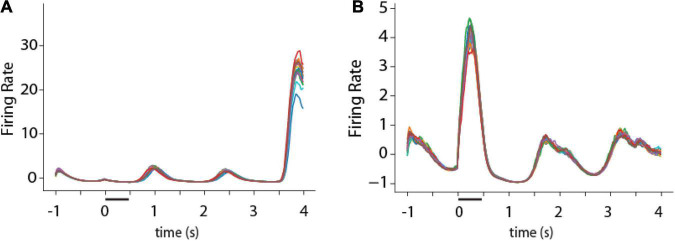
Activity of single RNN units in working memory task. **(A,B)** Peristimulus-time histogram of two responsive units from the fully trained stage of RNN in the ODR task. Traces of different colors represent firing rate in individual trials (*N* = 16 trials).

One consistent difference between RNN unit activity and firing rate of cortical neurons was that, for a given trained network, RNN unit activity tended to be much more stereotypical and reproducible from trial to trial. On the other hand, real neurons in the prefrontal cortex and other brain areas exhibit considerable variability from trial to trial during working memory (Qi and Constantinidis, 2012). This property can be appreciated in the PSTH of example RNN units (Figure 8). Except for brief periods of time during the response period of the unit depicted in Figure 8A, or the cue period in the unit depicted in Figure 8B, activity was virtually indistinguishable between different trials.

**FIGURE 9:**
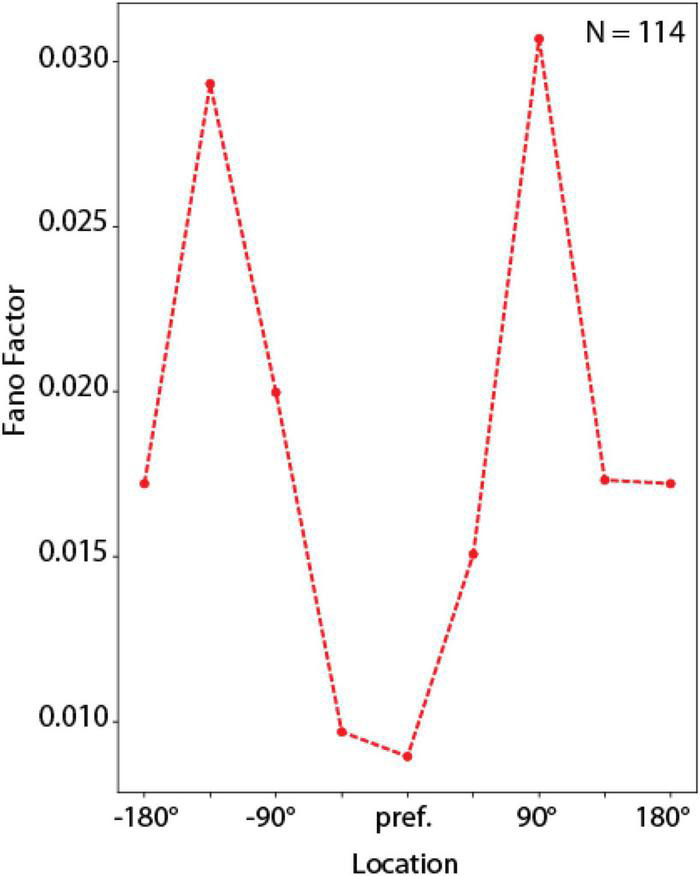
Fano factor analysis. Mean Fano factor values computed across units with delay period activity, for spatial locations relative to each unit’s preferred location. Curve represents average of *N* = 114 neurons, calculated over 16 correct trials for each location.

Trial-to-trial variability in brain neurons can be quantified formally with measures such as the Fano factor of spike counts (variance divided by the mean of the number of spikes at a given time). Use of the Fano factor for activity in a network that does not generate spikes is not entirely equivalent. Even if spikes are assumed to be generated by a Poisson process with mean rates equal to those achieved by the RNN, trial-to-trial spike counts in cortical neurons is determined by two types of variability: a network state reflected in mean firing rates, but also the stochasticity of the spike- generation process due to noisy inputs and probabilistic generation of action potentials. A much greater variability in spike counts would thus be expected in neurons than RNNs with equal mean rate. RNN unit activity can better be thought as an average of multiple neurons, and its Fano factor thus computed is not equivalent to that of individual neurons. Nonetheless, the Fano factor of RNN activity rates is still informative about the relative variability across conditions and the mean Fano factor computed over the delay period was therefore in the range of 0.02 (Figure 9), which is almost two orders of magnitude smaller than Fano factor of prefrontal cortical discharges (Qi and Constantinidis, 2012).

Despite this large quantitative difference, RNN captured another qualitative property of prefrontal cortical networks, variability during working memory that depends on the location of a remembered stimulus relative to the unit’s receptive field (Wimmer et al., 2014). In the context of the bump attractor model, following appearance of a stimulus at one location, the bump of activity in the population may drift randomly in either direction relative to the stimulus. If the stimulus appears at the flanks of the neuron’s receptive field, drifts in the direction of the peak would be expected to result in the increase in firing rate whereas drifts in the direction of the tail would be expected to result in lower firing rate. On the other hand, if the stimulus appeared at the peak of the receptive field, then any drift would be expected to result in lower firing rate, and therefore much less variability overall, from trial to trial. The same is true for locations at the tail of the unit’s tuning function. RNN units exhibited precisely the same pattern of variability, with an M-shaped Fano factor curve as a function of the unit’s tuning (Figure 9), similar to the pattern observed for the rate-behavior correlation. A 1-way Analysis of Variance revealed a significant effect of stimulus location on Fano factor [*F*_(7,904)_ = 5.05, *p* = 1.2 × 10^–5^]. This result, taken together with the rate-behavioral correlation results in Figure 7 indicates that, variability of the output of the RNN networks is governed by drifts of delay period activity. RNN units are activated in a predictable manner, as the activity of the network sweeps to represent different locations. The output of the network is governed by the relative activation of units representing different locations.

## Discussion

Artificial neural networks have been used widely over the past decade to solve computational problems as well as to uncover brain processes (LeCun et al., 2015). The success of convolutional neural networks in uncovering properties of neurons in the primate ventral visual pathway (Yamins and DiCarlo, 2016; Khaligh-Razavi et al., 2017; Rajalingham et al., 2018; Bashivan et al., 2019; Cadena et al., 2019) suggest that the same fundamental operations performed by the human brain are captured by artificial neural networks. This finding, in turn, allows the use of such networks as scientific models (Cichy and Kaiser, 2019; Saxe et al., 2021). Neuroscience principles have also been instructive for the design of more efficient networks and learning algorithms (Sinz et al., 2019). In addition to convolutional networks, other architectures have had practical applications in Neuroscience questions, for example to uncover neuronal spike dynamics, or encoding of elapsed time (Pandarinath et al., 2018; Bi and Zhou, 2020). The activity of the prefrontal cortex has been investigated successfully with Recurrent Neural Network frameworks, which capture many properties of the prefrontal cortex, including its ability to maintain information in memory and to perform multiple cognitive tasks, after training (Yang et al., 2019). We capitalized on these developments to study computational principles of working memory maintenance.

### Bump Attractor vs. Recurrent Neural Networks

In our current study, we performed a number of analyses in the same fashion as previous studies that have tied neurophysiological activity of neurons in the prefrontal cortex with behavior in working memory tasks (Wimmer et al., 2014; Barbosa et al., 2020). The architecture of the RNN network is different from biophysically inspired bump attractor models in that the connectivity footprint of each RNN unit is formed through training, rather than being hardwired based on the relative tuning of connected units. Nonetheless the output of the trained RNN network was determined by activity in the delay period of the task in a fashion that resembled the bump attractor models. Similarly, we saw that variability of RNN networks is governed by drifts of activity in the network. RNN units are activated in a predictable manner, as the activity of the network sweeps to represent different locations. The output of the network is governed by the relative activation of units representing different locations.

In a sense, the structure of the RNN resembles more that of the prefrontal cortex, which does not contain an orderly organization of neurons with precisely outlined connections (Constantinidis et al., 2001; Leavitt et al., 2017), unlike ring attractor models which simplify and idealize the organization of neurons and their synaptic connections. Other differences were also present, for example RNN units with different stimulus preference during the cue and delay periods (Figure 4), which again resembled more experimental results (Rao et al., 1999; Spaak et al., 2017) than their idealized, bump-attractor instantiation. Weight-updating is also in line with experimental findings of changes in synaptic connections observed in the primate prefrontal cortex (Zhou et al., 2014). Our results give credence to the idea that synaptic connections may be adjusted appropriately between prefrontal neurons, even absent a clear topographic organization and that the population of neurons may then behave like an attractor network even if activity of individual neurons does not appear perfectly canonical.

### Model Limitations

Despite the similarities between RNNs and prefrontal cortex, we do not wish to overstate their analogy. Some RNN instantiations maintained information in the delay period in a transient fashion, which is a documented property of RNNs trained to maintain information in short-term memory (Orhan and Ma, 2019). “Ringing” (oscillatory) dynamics were also much more prominent in the RNNs than the PFC data, though we should note such dynamics have been observed in other neural datasets, and arguments have been made of a significant role in working memory maintenance (Roux and Uhlhaas, 2014; Lundqvist et al., 2016; de Vries et al., 2020). We additionally relied on a small network of 256 units for these simulations, which greatly underestimates the complexity of the prefrontal cortex. Millions of neurons make up the real network, which is additionally organized in several subregions with distinct properties and capacity for plasticity (Riley et al., 2017, 2018), and encompasses areas beyond the prefrontal cortex (Jaffe and Constantinidis, 2021). Responses across trials were also highly stereotypical and lacked the variability present in prefrontal discharges. These examples illustrate that RNNs are not expected to be a precise replica of the brain. Nonetheless, the use of RNNs can reveal important computational principles of the brain.

Our study also did not examine directly opposing models of working memory, such as those relying on synaptic mechanisms. RNN networks allowing for forms of non-activity dependent plasticity have indeed shown ability to learn at least simple working memory tasks (Masse et al., 2019). We do not wish therefore to imply that our current study is definitive regarding the relative importance of spiking and non-spiking mechanisms (Masse et al., 2019).

### Summary of Insights and Outlook for Future Research

Our study demonstrates that persistent activity can be generated in a network of units whose activity represents a stimulus held in memory and whose structure of synaptic weights is determined by their relative preference for different stimuli. Our approach offers promise for understanding more complex working memory tasks are performed by neural circuits, including object working memory which requires representation of stimulus identity rather than spatial location; tasks that require manipulation of information in working memory; and tasks probing the capacity limitation of human working memory. Our approach offers a path toward identifying plausible neural mechanisms for these phenomena, which can then be probed with future experimental results.

## Data Availability Statement

The original contributions presented in the study are included in the article/supplementary material, further inquiries can be directed to the corresponding author/s.

## Author Contributions

XZ and CC designed the research. YX and YL performed the simulations. YX, YL, and XZ performed the analysis. YX, XZ, and CC wrote the manuscript. All authors contributed to the article and approved the submitted version.

## Conflict of Interest

The authors declare that the research was conducted in the absence of any commercial or financial relationships that could be construed as a potential conflict of interest.

## Publisher’s Note

All claims expressed in this article are solely those of the authors and do not necessarily represent those of their affiliated organizations, or those of the publisher, the editors and the reviewers. Any product that may be evaluated in this article, or claim that may be made by its manufacturer, is not guaranteed or endorsed by the publisher.
